# Ludwig’s Angina and Mandibular Osteomyelitis after Dental Extraction

**DOI:** 10.31662/jmaj.2018-0031

**Published:** 2018-11-12

**Authors:** Yuki Otsuka, Ko Harada, Yoshito Nishimura, Fumio Otsuka

**Affiliations:** 1 Department of General Medicine, Okayama University Graduate School of Medicine, Dentistry and Pharmaceutical Sciences, Okayama, Japan

**Keywords:** Ludwig’s angina, Osteomyelitis, Dental infection, Imaging

A 63-year-old Japanese man was admitted to our hospital owing to dysphagia, hoarseness, and jaw swelling (Figure 1A, B). He had undergone left lower wisdom-tooth extraction 3-weeks prior to admission. Contrast-enhanced computed tomography (CT) demonstrated submandibular cellulitis and abscess formation (Figure 1C). Magnetic resonance imaging (MRI) showed a low signal intensity lesion in the left mandible on the T1-weighted image (Figure 1D). These findings were consistent with Ludwig’s angina complicated with abscess and submandibular osteomyelitis. Blood cultures obtained on the day of admission showed negative results. Intravenous ampicillin/sulbactam was administered, and the symptoms improved. Ludwig’s angina, a severe cellulitis in submandibular space, is most commonly caused by a dental infection, especially in the second or third molar ^(1)^. Submandibular osteomyelitis is a severe complication after dental therapy; however, its diagnosis is often neglected because of its indeterminate symptoms ^(2)^. Furthermore, owing to the low sensitivity of CT in the diagnosis of osteomyelitis, MRI is essential when osteomyelitis is suspected.

**Figure 1.**
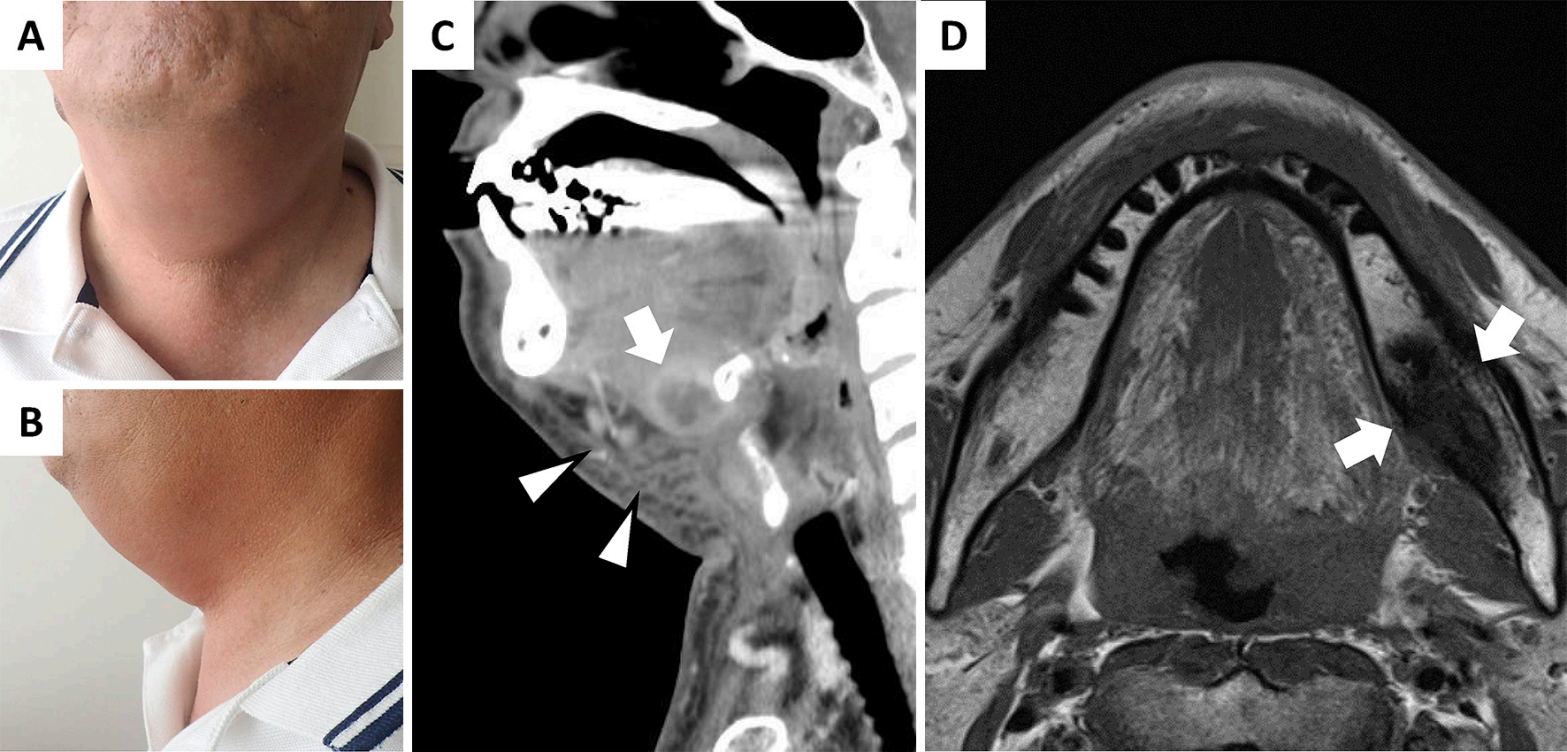
(A), (B) Physical examination revealed swelling and redness in the jaw. (C) Contrast-enhanced computed tomography (CT) demonstrated cellulitis in the patient’s jaw (*arrowheads*) and formation of an abscess (20-mm diameter) in front of the hyoid bone (*arrow*). (D) Magnetic resonance imaging (MRI) showed a low signal intensity lesion in the body of the left mandible on the T1-weighted image (*arrows*).

## Article Information

### Conflicts of Interest

None

### Author Contributions

YO wrote the manuscript. KH was an inpatient attending physician and edited the manuscript. YN edited the manuscript. FO supervised all the procedures.

### Ethical Statement

Informed consent was obtained from the patient.
